# The Role of Antioxidants in Skin Cancer Prevention and Treatment

**DOI:** 10.1155/2014/860479

**Published:** 2014-03-26

**Authors:** Aleksandar Godic, Borut Poljšak, Metka Adamic, Raja Dahmane

**Affiliations:** ^1^Department of Dermatology, Cambridge University Hospitals, Addenbrooke's Hospital, Hills Road, Cambridge CB2 0QQ, UK; ^2^Faculty of Health Studies, Zdravstvena pot 5, 1000 Ljubljana, Slovenia; ^3^Dermatology Centre Parmova, Parmova Street 53, 1000 Ljubljana, Slovenia

## Abstract

Skin cells are constantly exposed to reactive oxygen species (ROS) and oxidative stress from exogenous and endogenous sources. UV radiation is the most important environmental factor in the development of skin cancer and skin aging. The primary products caused by UV exposure are generally direct DNA oxidation or generation of free radicals which form and decompose extremely quickly but can produce effects that can last for hours, days, or even years. UV-induced generation of ROS in the skin develops oxidative stress when their formation exceeds the antioxidant defense ability. The reduction of oxidative stress can be achieved on two levels: by lowering exposure to UVR and/or by increasing levels of antioxidant defense in order to scavenge ROS. The only endogenous protection of our skin is melanin and enzymatic antioxidants. Melanin, the pigment deposited by melanocytes, is the first line of defense against DNA damage at the surface of the skin, but it cannot totally prevent skin damage. A second category of defense is repair processes, which remove the damaged biomolecules before they can accumulate and before their presence results in altered cell metabolism. Additional UV protection includes avoidance of sun exposure, usage of sunscreens, protective clothes, and antioxidant supplements.

## 1. Introduction

Ultraviolet radiation (UVR) is an essential risk factor for the development of premalignant skin lesions as well as of melanoma and nonmelanoma skin cancer. Skin cancer generally develops in the epidermis (the outermost layer of skin), so a tumor is usually clearly visible, which makes it easier to detect. There are various types of skin cancer. One main class is formed by the cutaneous melanocytes—melanoma. The other main types are basal cell carcinoma and squamous cell carcinoma, cancers of the epithelial cells. These carcinomas of the skin (basal cell and squamous cell carcinomas) are sometimes, collectively, called “nonmelanoma skin cancers.”

While exposure to UVR is the risk factor most closely linked to the development of skin cancer, other environmental factors (such as ionizing radiation, chronic arsenic ingestion, and immunosuppression) and genetic factors (such as family history, skin type, and genetic syndromes) also potentially contribute to carcinogenesis. UVR exposure appears to promote the induction of skin cancer by two mechanisms. The first involves direct mutagenesis of epidermal DNA, which promotes the induction of neoplasia. The second is associated with immune suppression, which allows the developing tumor to escape immune surveillance and grow progressively [1].

It is known that UVR exposure results in photochemical modification of the genetic material (DNA), but most of this damage is accurately and efficiently repaired by the cell. However, if the amount of damage is too great, some of the alterations to the DNA may remain as permanent mutations. DNA absorbs UV light, and the absorbed energy can break bonds in the DNA. Most of the DNA breakages are repaired by proteins present in the cell nucleus, but unrepaired genetic damage of the DNA can lead to skin cancers. As already mentioned previously, solar UVR induces a variety of photoproducts in DNA, including cyclobutane-type pyrimidine dimers, pyrimidine-pyrimidone (6–4) photoproducts, thymine glycols, cytosine damage, purine damage, DNA strand breaks, and DNA-protein crosslinks. It has been proposed that if unrepaired damage occurs to regulatory genes (e.g., tumor suppressor genes), this may be involved in the process of carcinogenesis. In this context, mutations to and activation of genes may be important. Other responses likely to result from UVR exposure of cells include increased cellular proliferation, which could have a tumor-promoting effect on genetically altered cells, as well as changes in components of the immune system present in the skin [2].

Solar radiation was tested for carcinogenicity in a series of exceptional studies in mice and rats. Large numbers of animals were studied, and well-characterized benign and malignant skin tumors developed in most of the surviving animals. Although the reports are deficient in quantitative details, the results provide convincing evidence that sunlight is carcinogenic for the skin of animals [3]. Although DNA damage due to reactive oxygen species formation is not a rare event since it is estimated that human cell sustains an average of 10^5^ oxidative hits per day due to cellular oxidative metabolism [4], DNA is functionally very stable, so that the incidence of cancer is much lower than one would expect, taking into account the high frequency of oxidative hits.

It seems that in rapidly dividing epithelium, such as the epidermis, nuclear damage triggered by some xenobiotics may not be so important because of the constant introduction of new healthy cells, whereas a DNA mutation has a much higher probability to become fixed to a transformed phenotype in tissues (e.g., liver) with slow cell turnover [5]. This may explain at least in part why the absolute number of clinically well-recognizedhuman skin carcinogens is so small. The specific mutations needed to activate an oncogene would be rarer. The probability of mutating five genes needed for cancer formation, such as an oncogene and both alleles of two particular tumor suppressor genes, is at best 10^−20^. With 10^6^ proliferating keratinocytes per cm^2^ in human skin, and ~1 cm^2^ exposed, less than one person in 10^10^ would develop a tumor. However, clonal expansion increases by 1,000-fold the number of targets for the next mutation and increases the probability of tumor formation. It is widely believed that cancer development inhumans and laboratory animals is caused by sequential mutations and clonal outgrowth of somatic cells.

Most important oxidative damage prevention mechanisms include antioxidative enzymatic and nonenzymatic defenses as well as repair processes. But the problem arises with age, when endogenous antioxidative mechanisms and repair processes do not work anymore in the effective way. The identification of free radical reactions as initiators and promoters of the cancer process implies that interventions aimed at limiting or inhibiting these factors should be able to reduce the rate of cancer incidence. There still remains the answer regarding controversial data on the use of synthetic antioxidants in cancer prevention and treatment.

## 2. Skin Antioxidant Defenses

Although the skin possesses an elaborate antioxidant defense system to deal with oxidative stress, excessive and chronic exposure to UV light or other oxidizing agents (e.g., cigarette smoke) can overwhelm the cutaneous antioxidant and immune response capacity, leading to oxidative damage and immunotoxicity, premature skin aging, and skin cancer.

A biological antioxidant has been defined as any substance that when present at low concentrations compared to those of an oxidizable substrate significantly delays or prevents oxidation of that substrate [6]. Antioxidant functions are associated with lowering oxidative stress, DNA damage, malignant transformation, and other parameters of cell damage* in vitro* as well as epidemiologically with lowered incidence of certain types of cancer and degenerative diseases. Antioxidants attenuate the damaging effects of reactive oxygen species (ROS) and can impair and/or reverse many of the events that contribute to epidermal toxicity and disease. However, increased or prolonged free radical action can overwhelm ROS defense mechanisms, contributing to the development of cutaneous diseases, disorders, and skin cancer. The two main categories of antioxidant defenses are those whose role is to prevent the generation of ROS and those that intercept any radicals that are generated [7]. The defense system exists in aqueous and membrane compartments of cells and can be enzymatic and nonenzymatic. A second category of natural antioxidants are repair processes, which remove the damaged biomolecules before they accumulate to cause altered cell metabolism or viability [7].

The skin is equipped with a network of protective antioxidants. They include enzymatic antioxidants such as glutathione peroxidase, superoxide dismutase and catalase, and nonenzymatic low-molecular-weight antioxidants such as vitamin E isoforms, vitamin C, glutathione (GSH), uric acid, and ubiquinol [8]. Various other components present in skin are potent antioxidants including ascorbate, uric acid, carotenoids, and sulfhydrils. Water-soluble antioxidants in plasma include glucose, pyruvate, uric acid, ascorbic acid, bilirubin, and glutathione. Lipid-soluble antioxidants include alpha-tocopherol, ubiquinol-10, lycopene, *β*-carotene, lutein, zeaxanthin, and alpha-carotene. In general, the outer part of the skin, the epidermis, contains higher concentrations of antioxidants than the dermis [9, 10]. In the lipophilic phase, *α*-tocopherol is the most prominent antioxidant, while vitamin C and GSH have the highest abundance in the cytosol. On molar basis, hydrophilic nonenzymatic antioxidants including L-ascorbic acid, GSH, and uric acid appear to be the predominant antioxidants in human skin [11]. Their overall dermal and epidermal concentration are more than 10- to 100-fold greater than those found for vitamin E or ubiquinol.

The antioxidant capacity of the human epidermis is far greater than that of dermis. This was demonstrated in the studies by Shindo et al. [9, 10] where enzymatic and nonenzymatic antioxidants in human epidermis and dermis from six healthy volunteers undergoing surgical procedures were measured. A similar study was done by Shindo et al. [8] where enzymatic and nonenzymatic antioxidants in epidermis and dermis of hairless mice were compared. Catalase, glutathione peroxidase, and glutathione reductase were higher in epidermis than dermis. Lipophilic antioxidants (alpha-tocopherol, ubiquinol 9, and ubiquinone 9) and hydrophilic antioxidants (ascorbic acid, dehydroascorbic acid, and glutathione) were also higher in epidermis than in dermis. The stratum corneum (SC) was found to contain both hydrophilic and lipophilic antioxidants. Vitamins C and E (both *γ*- and *α*-tocopherols) as well as GSH and uric acid were found to be present in the SC [12]. Surprisingly, they were not distributed evenly, but in gradient fashion, with low concentrations on the outer layers and increasing concentrations toward the deeper layers of the SC. This phenomenon may be explained by the fact that O_2_ partial pressure is higher in the upper SC, which already causes a mild oxidative stress resulting in the partial depletion of antioxidants.

Taken together, all the major antioxidant enzymes are present in skin but their role in protecting cells against oxidative damage generated by UVR has not been elucidated. In response to the attack of ROS, the skin has developed a complex antioxidant defense system including, among others, the manganese-superoxide dismutase (MnSOD). The study of Poswig et al. [13] revealed that adaptive antioxidant response of manganese-superoxide dismutase following repetitive UVA irradiation can be induced. The authors provide evidence for the increasing induction of MnSOD upon repetitive UVA irradiation that may contribute to the effective adaptive UVA response of the skin during light hardening in phototherapy. The study of Fuchs et al. [5] on mouse skin showed that acute UV exposures lead also to changes in glutathione reductase and catalase activity in mouse skin but to insignificant changes in superoxide dismutase and glutathione peroxidase. The study of Sander et al. [14] confirmed that chronic and acute photodamage are mediated by depleted antioxidant enzyme expression and increased oxidative protein modifications.

## 3. The Importance of Antioxidants in Decreasing ROS Formation and Skin Cancer Prevention

The only protection of our skin against UVR is its endogenous protection (melanin and enzymatic antioxidants) and antioxidants we consumed with the food (vitamin A, C, E, etc.). Dietary antioxidants thus play a major role in maintaining the homeostasis of the oxidative balance. Vitamin C (ascorbic acid), vitamin E (*α*-tocopherol), beta-carotene, and other micronutrients such as carotenoids, polyphenols, and selenium have been evaluated as antioxidant constituents in the human diet. UVR exposure affects the skin antioxidants. Ascorbate, GSH, SOD, catalase, and ubiquinol are depleted in UV-B exposed skin, both dermis and epidermis. Levels of electron paramagnetic resonance (EPR) detectable ascorbyl radicals rise on UV exposure of skin. Studies of cultured skin cells and murine skin* in vivo* have indicated that UVR-induced damage involves the generation of ROS and depletion of endogenous antioxidant systems [15]. For example, in the study by Shindo et al. [8], enzymatic and nonenzymatic antioxidants in epidermis and dermis and their responses to ultraviolet light of hairless mice were compared. After irradiation epidermal and dermal catalase and superoxide dismutase activities were greatly decreased. *α*-Tocopherol, ubiquinol 9, ubiquinone 9, ascorbic acid, dehydroascorbic acid, and reduced glutathione decreased in both epidermis and dermis by 26–93%. Oxidized glutathione showed a slight, nonsignificant increase. Many other studies confirmed that acute exposure of human skin to UVR* in vivo* leads to oxidation of cellular biomolecules that could be prevented by prior antioxidant treatment. There have been many studies performed where different antioxidants or combinations of antioxidants and different phytochemicals were tested in order to find evidence against ROS-induced damage. The outcomes of the studies examining the influence of exogenous antioxidants on the photoaging or damage protective effects, which are relevant for clinical practice, were in details presented elsewhere by Pandel et al. [16] and Poljsak et al. [17].

## 4. Vitamin C

Oral vitamin C supplements (500 mg/day) were taken by 12 volunteers for 8 weeks resulting in significant rises in plasma and skin vitamin C content [15]. Supplementation had no effect on the UVR-induced erythemal response. The skin malonaldehyde content was reduced by vitamin C supplementation, but, surprisingly, reductions in the skin content of total glutathione and protein thiols were also seen. Authors speculate that this apparently paradoxical effect could be due to regulation of total reductant capacity by skin cells, such that vitamin C may have been replacing other reductants in these cells.

Ascorbic acid was a photoprotectant in clinical human UV studies at doses just above the minimal erythema dose (MED). An opaque cream containing 5% ascorbic acid did not induce dermal sensitization in 103 human subjects. A product containing 10% ascorbic acid was nonirritant in a 4-day minicumulative patch assay on human skin and a facial treatment containing 10% ascorbic acid was not a contact sensitizer in a maximization assay on 26 humans [15]. Many other studies have found that vitamin C can increase collagen production, protect against damage from UVA and UVB rays, correct pigmentation problems, and improve inflammatory skin conditions (reviewed in [16–18]).

## 5. Vitamin E

Skin exposure to UV and ozone alone and in combination resulted in a significant potentiation of the UV-induced vitamin E depletion [19], which means that vitamin E is efficiently quenching ROS during UVR skin exposure. Depletion of vitamin E is one of the earliest oxidative stress markers in human skin exposed to UVR and other environmental stress [20]. One study showed that the number of sunburn to cells was decreased by treatment with the antioxidant tocopherol and may result from both direct protection from free radicals and indirect protection by means of increased epidermal thickness [21]. Additionally, Packer and Valacchi [19] showed that vitamin E has skin barrier-stabilizing properties. Vitamin E provides protection against UV-induced skin photodamage through a combination of antioxidant and UV absorptive properties. Topical application of alpha-tocopherol on mouse skin inhibits the formation of cyclobutane pyrimidine photoproducts. However, topically applied alpha-tocopherol is rapidly depleted by UVB radiation in a dose-dependent manner [22].

## 6. *β*-Carotene

*β*-carotene is a major constituent of commercially available products administered for systemic photoprotection. *β*-carotene supplements are frequently used as so-called oral sun protectants, but studies proving a protective effect of oral treatment with *β*-carotene against skin responses to sun exposure are scarce and conflicting results have been reported [23]. Studies on the systemic use of *β*-carotene provide evidence that 15–30 mg/d over a period of about 10–12 weeks produces a protective effect against UV-induced erythema. Similar effects have been attributed to mixtures of carotenoids or after long-term intake of dietary products rich in carotenoids. Supplementation with carotenoids contributes to basal protection of the skin but is not sufficient to obtain complete protection against severe UV irradiation [23]. Studies showed that the efficacy of *β*-carotene in systemic photoprotection depends on the duration of treatment and on the dose [23]. For successful intervention, treatment with carotenoids is needed for a period of at least ten weeks [24]. A study by Stahl et al. [25] was performed where carotenoids and tocopherols antioxidant effect was investigated against scavenging of ROS generated during photooxidative stress. It was investigated whether antioxidant oral supplementation may protect the skin from UV-induced erythema. The antioxidants used in this study provided protection against erythema in humans and may be useful for diminishing sensitivity to UV light. Heinrich et al. [26] additionally compared the erythema protective effect of beta-carotene (24 mg/d from an algal source) to that of 24 mg/d of a carotenoid mix consisting of the three main dietary carotenoids, beta-carotene, lutein, and lycopene (8 mg/d each). A randomized, placebo-controlled clinical trial on the efficacy of oral *β*-carotene (50 mg/day over 5 years) in prevention of skin cancer in patients with recent nonmelanoma skin cancer showed no significant effect of *β*-carotene on either number or time of occurrences of new nonmelanoma skin cancer [27]. In a separate trial among healthy men, 12 years of supplementation with *β*-carotene (50 mg on alternate days) produced no reduction of the incidence of malignant neoplasms, including nonmelanoma skin cancer [28]. It must be pointed out that these intervention trials were conducted with patients whose skin cancer was primarily UV induced and it remains to be seen whether antioxidants are clinically effective in prevention of cutaneous chemocarcinogenesis [29]. Although the photoprotective effects of beta-carotene are thought to originate from its antioxidant properties, some studies documented prooxidant effects of beta-carotene.

## 7. Retinoids

A study was done to compare the effects of dietary administration of a vitamin A drug (13-cis-retinoic acid) to the natural form of vitamin A (retinyl palmitate). Female mice were administered a chemical carcinogen to evaluate the incidence and severity on mouse skin tumor promotion. The results showed that retinyl palmitate inhibited the number and weight of tumors, whereas 13-cis-retinoic acid resulted in a decrease in weight but not in number of tumors promoted [30]. In another study, tumors were chemically induced in a group of Swiss mice over a 23-week period. The topical application of 13-cis-retinoic acid was compared to natural vitamin A (retinyl palmitate). This study showed that both retinyl palmitate and 13-cis-retinoic acid inhibited the development of skin papillomas and also had a marked effect on skin cancers [31].

## 8. Coenzyme Q10

It was recently reported that coenzyme Q10 protects against oxidative stress-induced cell death and enhances the synthesis of basement membrane components in dermal and epidermal cells [32]. Coenzyme Q10 (CoQ10) was reported to reduce ROS production and DNA damage triggered by UVA irradiation in human keratinocytes* in vitro*. Further, CoQ10 was shown to reduce UVA-induced MMPs in cultured human dermal fibroblasts [33]. It was reported that it is considered that CoQ10 appears to have also a cutaneous healing effects* in vivo* [34].

## 9. Glutathione 

In cell culture models using human skin cells, it has been clearly shown that glutathione depletion leads to a large sensitization to UVA (334 nm, 365 nm) and near-visible (405 nm) wavelengths as well as to radiation in the UVB (302 nm, 313 nm) [35, 36]. There is a direct correlation between the levels of sensitization and cellular glutathione content. Additional evidence that glutathione is a photoprotective agent in skin cells is derived from experiments which have demonstrated that glutathione levels in both dermis and epidermis are depleted by UVA treatment [37].

## 10. Green Tea

*In vitro* and* in vivo* animal and human studies suggest that green tea polyphenols are photoprotective in nature and can be used as pharmacological agents for the prevention of solar UVB light-induced skin disorders including photoaging, melanoma, and nonmelanoma skin cancers after more clinical trials in humans. Topical treatment or oral consumption of green tea polyphenols (GTP) inhibits chemical carcinogen- or UV radiation-induced skin carcinogenesis in different laboratory animal models. Topical application of GTP prior to exposure of UVB protects against UVB-induced local as well as systemic immune suppression in laboratory animals, which was associated with the inhibition of UVB-induced infiltration of inflammatory leukocytes [38]. Another study of Vayalil et al. [39] demonstrated that topical application of green tea polyphenols reduced UVB-induced oxidation of lipids and proteins and depletion of antioxidant enzymes. Other protective effects include the reduced production of ROS and lipid peroxidation products, a reduced depletion of Langerhans cells and of endogenous antioxidant systems as reported by Afaq and Mukhtar [40].

## 11. Conclusions

Skin DNA molecules are constantly “bombarded” by ROS originating from endogenous processes as well as from environmental agents and from radiation sources. Antioxidants might act by quenching free radicals and by enhancing the DNA enzyme repair systems through a posttranscriptional gene regulation of transcription factors [41]. The repair capacity of human skin cells therefore directly relates to the probability of initiation of the carcinogenesis process and eventually tumor formation. Evidence is accumulating that dietary changes and special nutrients may help to reduce oxidative stress and free radical formation and thereby slow down the skin damage process. Exogenous antioxidants like vitamins C and E and many others cannot be synthesized by the human body and must be taken up by the diet. Since the effectiveness of endogenous antioxidant system is diminished during aging, the exogenous supplementation of antioxidants might be a protective strategy against age-associated skin oxidative damage. It can be concluded that oxidative stress is a problem of skin cells and endogenous as well as exogenous antioxidants could play an important role in decreasing it.

However, it is important to pretreat the skin with antioxidants before sun exposure. Animal and human studies have convincingly demonstrated pronounced photoprotective effects of “natural” and synthetic antioxidants when applied topically before UVR exposure. No significant protective effects of melatonin or the vitamins when applied alone or in combination were obtained when antioxidants were applied after UVR exposure. UVR-induced skin damage is a rapid event, and antioxidants possibly prevent such damage only when present in relevant concentration at the site of action at the beginning and during oxidative stress [42]. Treatment of the skin with antioxidants after the damage was caused by UVR might cause additional harmful effects on cell cycle control and apoptosis process. Antioxidants may thus have dichotomous activities with respect to carcinogenesis, namely, suppressing carcinogenesis by preventing oxidative damage to DNA [43] and promoting carcinogenesis by allowing survival of cells that are metabolically impaired (e.g., in altered matrix environments). Besides, the photoprotective effects of antioxidants are significant when applied in distinct mixtures in appropriate vehicles. According to Stahl et al. [23], endogenous photoprotection is complementary to topical photoprotection, and these two forms of prevention clearly should be considered mutually exclusive. The most important strategy to reduce the risk of sun UV radiation damage is to avoid the sun exposure and the use of sunscreens. The next step is the use of exogenous antioxidants orally or by topical application and interventions in preventing oxidative stress and in enhanced DNA repair.
