# Corrigendum to “The Role of Antioxidants in Skin Cancer Prevention and Treatment”

**DOI:** 10.1155/2020/1969760

**Published:** 2020-08-31

**Authors:** Aleksandar Godić, Borut Pojšak, Metka Adamič, Raja Gošnak Dahmane

**Affiliations:** ^1^Department of Dermatology, Cambridge University Hospitals, Addenbrooke's Hospital, Hills Road, Cambridge CB2 0QQ, UK; ^2^Faculty of Health Studies, Zdravstvena pot 5, 1000 Ljubljana, Slovenia; ^3^Dermatology Centre Parmova, Parmova Street 53, 1000 Ljubljana, Slovenia

The article titled “The Role of Antioxidants in Skin Cancer Prevention and Treatment” [1] was found to contain a substantial amount of overlapping material with the authors' previously published article [2].

Significant text overlap was also identified with the following sources:

[3] Santosh K. Katiyar, “Skin photoprotection by green tea: antioxidant and immunomodulatory effects”, Current Drug Targets - Immune, Endocrine & Metabolic Disorders, 2003. 10.2174/1568008033340171 (cited as reference 38 in the original article).

[4] *An article posted in Life Extension Magazine*, January 2003 (https://www.lifeextension.com/magazine/2003/1/cover_skinaging/Page-01). (not cited in the original article).

[5] McArdle F, Rhodes LE, Parslew R, Jack CI, Friedmann PS, Jackson MJ, “UVR-induced oxidative stress in human skin in vivo: effects of oral vitamin C supplementation”, 2002. 10.1016/S0891-5849(02)01042-0 [5] (cited as reference 15 in the original article).

The authors apologize for this overlap.
